# Micronutrients and the Immune System: Some Is Good but We Need to Know More

**DOI:** 10.3390/nu13020285

**Published:** 2021-01-20

**Authors:** Henry J. Thompson

**Affiliations:** Cancer Prevention Laboratory, Colorado State University, Fort Collins, CO 80523, USA; henry.thompson@colostate.edu; Tel.: +1-970-491-7748; Fax: +1-970-491-3542

Defining the role of nutrients in disease prevention and control has entered a renaissance because of both breakthroughs in studying the gut associated microbiome [1,2] and developments in re-understanding the role that immune system plays in the prevention and control of communicable and non-communicable diseases [3]. Progress has occurred not only within these respective areas but at their interface [4,5]. Moreover, these advances need to be considered in the broader framework of the importance of whole foods in health and disease [6]. The fact that whole foods and ingredients in processed food exert beneficial and detrimental effects on the host that are mediated, at least in part, by the gut associated microbiome, that in turn impact the immune system, underscores the importance of understanding the food-microbiome-immune axes in the prevention and control of disease. The “encyclopedic review”, authored by Gombart, Pierre, and Maggini, is highly timely and an invaluable resource for the field. What is particularly helpful is the dissection of the effects of micronutrients on various components of the innate and adaptive arms of the immune system. However, the authors bury an important lede, namely that we really don’t know whether micronutrients exert effects on the immune system that are causally important in disease prevention and control when the intake of those micronutrients exceed levels of consumption that are above those associated with clinical deficiency, nor do we understand how the form of dietary micronutrient intake, i.e., food versus supplement, impacts the effects micronutrients exert on these axes [7]. 

From the earliest pen strokes in their review, Gombart, Pierre, and Maggini appropriately remind the reader of the evidence that clinical deficiencies of specific micronutrients impair immune responsiveness [7]. A classic example that is cited is the role of vitamin C in scurvy. Here the evidence for causality is clear cut. There are two global situations to which these findings are particularly salient. The most obvious is in regions of the world where observable micronutrient deficiencies are manifest. In these circumstances there have been extensive efforts to improve micronutrient status across the intervention spectrum, including whole food enrichment via crop breeding and selection, e.g., Harvest Plus; induced changes in micronutrient composition of a food via genetic engineering, e.g., golden rice; the deployment of micronutrient fortified ingredients for use during food preparation; and the use of nutrient supplements whether in a powdered or tablet form. The second situation is in the arena of “hidden hunger” i.e., the inadequate consumption of micronutrients frequently in the face of adequate or excess intake of calories, which is prevalent in both economically developing and developed countries [8].

The role of specific micronutrients and of various combinations are painstakingly documented in [7], although with increasing complexity of the interactions reported, it becomes less clear that the evidence presented meets criteria considered necessary for establishment of causal relations [9]. It is at this point that we enter of realm of slippery slopes. The real challenges in understanding the role of micronutrients in determining immune function are not under conditions of micronutrient clinical deficiency, but rather when micronutrient intake is suboptimal. Moreover, evidence that supplementation with micronutrients, above levels that meet Recommended Dietary Intakes, bolsters immune responsiveness in the absence of adverse outcomes is not compelling, especially when considered through the lens of causality. The harsh reality is that with the emerging demand for precision nutrition [10], which rightly recognizes individual variations in required levels of various nutrients, the establishment of causality will be difficult. Whether hypothesis driven or data driven approaches to scientific inquiry are used, the complex interactions among nutrients and individual variations in the amount of each nutrient that achieves maximal benefit will require large sample sizes and still make causal inference difficult. The temptation will be to presume biological benefit in the absence of strong evidence of causality. Significant problems can arise from such presumptions; they include increased risk of adverse effects of micronutrient supplements, many of which have been reported [11], as well as potential for wasting consumer resources on purchases that afford no demonstrable benefit. In addition, the skepticism of the medical community about the importance of nutrition in disease prevention and control will remain unless the underlying science is documented to meet the criteria of causality, as is common for many drugs dispensed to patients [12].

Food-gut microbiome-immune system interactions are an unquestionable focus of the recently released National Institutes of Health (USA) Nutrition Research Strategy [13,14]. The absence of this consideration in [7] is conspicuous but expected since this is largely an unplowed field that is rich in opportunities for discovery. Several potential areas of focus are offered for those interested in studying the role of micronutrients on the immune system as affected by the gut associated microbiome: (1) which research strategy(s) opens the door to the assessment of causality, i.e., hypothesis or data driven; (2) are there differences in response to the same daily intake of micronutrients when they are consumed as components of food versus as a nutrient supplement; and (3) what are the pharmacokinetic and pharmacodynamic underpinnings of the effects observed. 

A principal focus of the Gombart, Pierre, and Maggini review is directed to infectious diseases. While this is very appropriate, it must also be considered that 60% of deaths globally are due to chronic diseases in which the immune system, as well as the gut associated microbiome, play a significant role [15]. Whether or not micronutrient mediated effects on the immune system play similar roles in communicable and non-communicable disease processes will need to be clarified. As mentioned above, it is also important to keep in mind the case that has been made cyclically over the decades against micronutrient supplementation and the many examples of adverse effects that have been reported [11]. Thus, the review of Gombart, Pierre, and Maggini, while authoritative and broad in scope, is but the first chapter in an untold story whose conclusion must provide definitive guidance about micronutrients and disease risk and control that “Do No Harm”.
